# Tai Chi Chuan exercise related change in brain function as assessed by functional near–infrared spectroscopy

**DOI:** 10.1038/s41598-019-49401-9

**Published:** 2019-09-13

**Authors:** Hui Xie, Ming Zhang, Congcong Huo, Gongcheng Xu, Zengyong Li, Yubo Fan

**Affiliations:** 1 School of Biological Science and Medical Engineering, Beihang University, Beijing, China; 2 Beijing Advanced Innovation Centre for Biomedical Engineering, Beihang University, Beijing, China; 3 Department of Biomedical Engineering, Faculty of Engineering, Hong Kong Polytechnic University, Kowloon, Hong Kong, SAR P.R. China; 4 Beijing Key Laboratory of Rehabilitation Technical Aids for Old–Age Disability, National Research Center for Rehabilitation Technical Aids Beijing, Beijing, 100176 China; 5 Key Laboratory of Rehabilitation Aids Technology and System of the Ministry of Civil Affairs, Beijing, 100176 China

**Keywords:** Near-infrared spectroscopy, Cognitive neuroscience

## Abstract

Early studies have shown that Tai Chi Chuan (TCC) contributes to the rehabilitation of cognitive disorders and increases blood oxygen concentration levels in the parietal and occipital brain areas; however, the mechanism of TCC training on brain function remains poorly understood. This study hypothesize that TCC has altered brain function and aims to explore the effects of TCC on functional connection and effective connection of the prefrontal cortex (PFC), motor cortex (MC), and occipital cortex (OC). The participants were 23 experienced Chen–style TCC practitioners (TCC group), and 32 demographically matched TCC–naive healthy controls (control group). Functional and effective connections were calculated using wavelet–based coherence analysis and dynamic Bayesian inference method, respectively. Results showed that beyond the intensity of activity in a particular cortical region induced by TCC, significant differences in brain activity and dynamic configuration of connectivity were observed between the TCC and control groups during resting and movement states. These findings suggested that TCC training improved the connection of PFC, MC and OC in myogenic activity, sympathetic nervous system, and endothelial cell metabolic activities; enhanced brain functional connections and relayed the ability of TCC to improve cognition and the anti–memory decline potential.

## Introduction

Brain functions in the elderly, in terms of memory, reasoning, balance, coordination, spatial perception, and imagination, often decline with aging^1^. Degradation of brain function may greatly affect the health and autonomy of the elderly and thus lead to cognitive impairments and even dementia and stroke^2^. With the continuous aging of society, brain function degradation reduces the quality of life of the elderly and causes serious social burden. Thus, effectively preventing brain function degradation in the elderly is an urgent problem that must be solved.

Tai Chi Chuan (TCC) is a typical mind–body and low–intensity aerobic exercise that involves cognitive training and movement meditation and has positive associations with physiological and psychological conditions^3^. TCC has metabolic equivalents estimated between 1.5 and 4.0. This aerobic intensity overlaps with brisk walking which has been demonstrated to contribute to the prevention of cognitive decline^4^, and rehabilitation of dementia^5^ and stroke^6^; notably, TCC has been observed to improve power, balance, memory and attention after 6 months^7^. Moreover, TCC practice can affect the brain prefrontal structure and function^8^ and improve memory^9^, as observed by functional magnetic resonance imaging (fMRI). The parietal and occipital cortices in TCC practitioners were found to have thickened through the same method^8,10^. Electroencephalo–graph (EEG) showed significant theta activities in the fronto–central and centro–parietal cortical areas in TCC practitioners^11^ and TCC have been shown to reduce anxiety and depression^12^. However, the mechanism of the effects of TCC training on brain function remains poorly understood, especially in real–time body movements. Therefore, it is necessary to study the change in brain function related to TCC movement state.

Exploring the effect of TCC on hemodynamic modulation in body movements has become possible with the development of functional near–infrared spectroscopy (fNIRS) technique. fNIRS is a well–developed technology which has been used to get signals from prefrontal and motor cortex very effectively^13^; that measures the concentration of oxygenated hemoglobin (HbO_2_) and deoxygenated hemoglobin (HHb) in the cortical layer of the brain^14^. Compared with other non–invasive brain detection techniques such as fMRI and EEG, the main advantages of fNIRS are that it can detect the changes in HbO_2_ and HHb levels in the microcirculation of brain tissues with a relatively good spatial and temporal resolution. Moreover, portable fNIRS equipment is safe, convenient, inexpensive, and less restrictive for the movement^15,16^, is suitable for monitoring monitor brain functions of TCC practitioners during movement states. While NIRS could be affected by systemic physiological noise such as the interference of heart and respiration components^17,18^, it is possible to filter out such physiological noise based on the relative high sample rate of fNIRS devices (greater than 9.6 Hz)^17^. fNIRS has been widely used in brain function research and neuroimaging^19^, and the results have been consistent with those of fMRI^20^. Furthermore, fNIRS has been effective used in brain–computer interfacing because it is easily wearable and has good spatial resolution^21^.

Human hemodynamic signals have strong time–frequency characteristics. Spontaneous cerebral oxygenation signals have different physiological sources in various frequency intervals^22,23^. Five frequency intervals corresponding to different physiological sources have been identified in our previous studies^24,25^: I, 0.6–2 Hz II, 0.145–0.6 Hz; III, 0.052–0.145 Hz; IV, 0.021–0.052 Hz, and V, 0.0095–0.021 Hz. The I and II frequency intervals, represented the systemic fluctuations, which reflected the synchronization of cardiac and respiratory activities in the cerebral regions^26^, whereas the III to V frequency intervals were regarded as myogenic, neurogenic and endothelial cell metabolic activities^27,28^. Changes in brain areas and the temporal correlations of these changes can be measured by multichannel fNIRS instruments, thereby rendering fNIRS–based “functional or effective” connectivity^29,30^. Functional connection (FC) is defined as the correlation between different neurophysiological activities, whereas effective connection (EC) refers to the influence of one neural system on the other neural systems, both are parameters for evaluating brain function^31^. FC is simply a statistically quantifiable and observable phenomenon, similar to relevance and consistency. EC is a model parameter that explains the dependence of emergence^32^. FC mainly evaluates the degree of coordination between different brain regions based on wavelet phase coherence (WPCO), whereas EC determines the direction of the brain segment based on coupling strength and direction.

The prefrontal cortex (PFC), motor cortex (MC), and occipital cortex (OC) are involved in the motor behavioral activities in the body. Among these, PFC plays an important role in cognitive control^33,34^. MC is located on both sides of the central ditch of the brain and mainly controls the movement of the human body through spatial sensation and movement planning^35^. Lastly, within the OC is the visual cortex^36^, which is related to visual perception^37^. Research has shown that exercise could provide a simple means to maintain brain function and promote brain plasticity^38,39^. The study hypothesize that TCC has altered brain function. Thus, this study measured the resting state and TCC movement state of ordinary people and long–term TCC practitioners, in order to explore the mechanism action of TCC and the change of brain function in the PFC, MC and OC of TCC training by using fNIRS. The results will provide insights into the mechanism of TCC training and its effect on brain function improvement and will contribute to the improvement of TCC practice strategies.

## Results

### Wavelet amplitude

**Figure 1:**
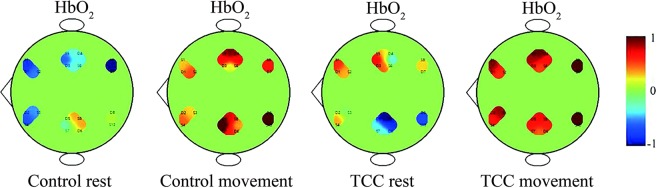
Example of the intensity or activation of the cerebral cortex in the resting state of both groups and movement state of the TCC group. Cool color represents the activation in the six brain regions, and the warm color represents higher activation than the cool–colored regions.

**Figure 2:**
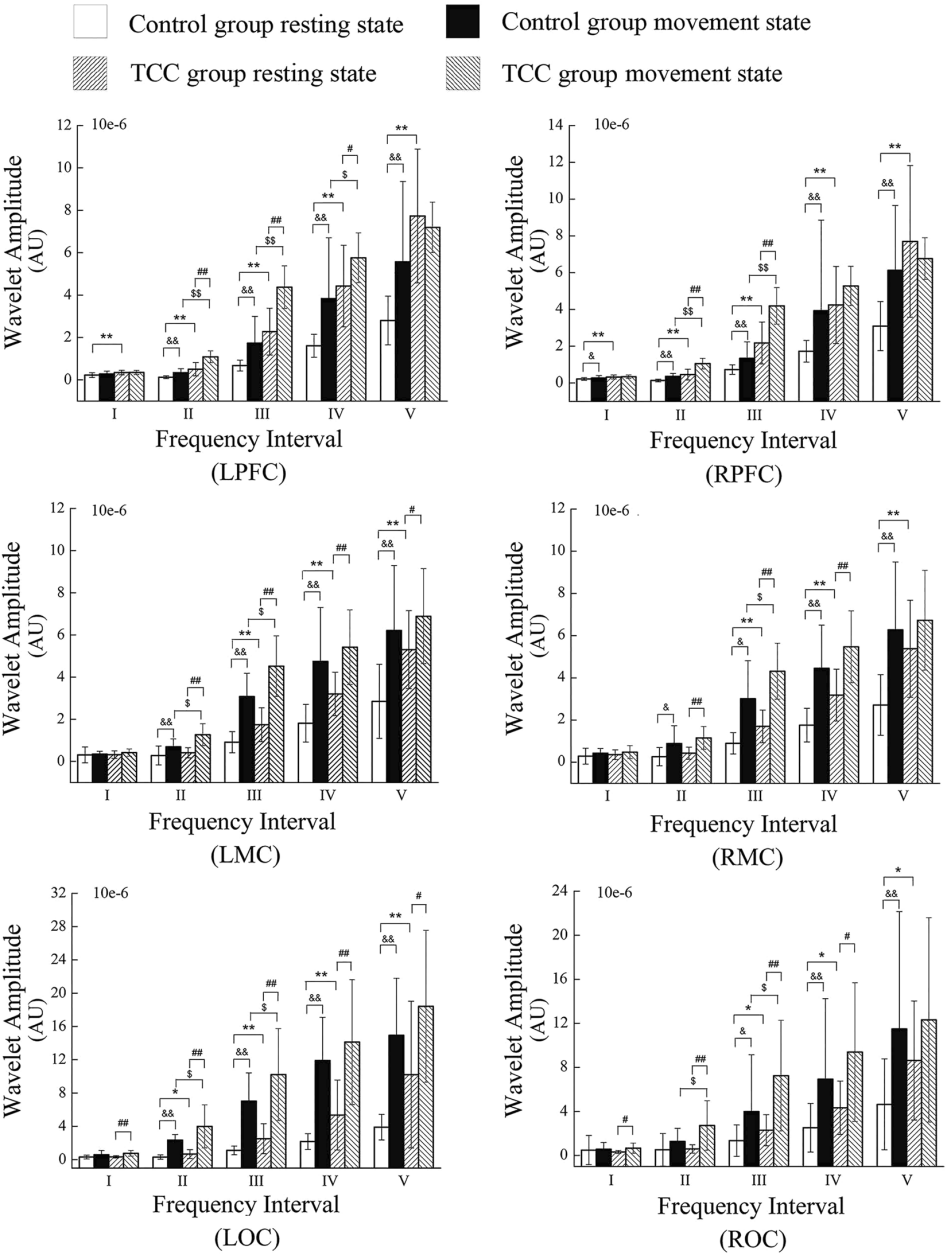
Comparison of WA values in different regions at frequency intervals I–V. *(*p* < 0.05) and **(*p* < 0.001) indicate significant difference between the control and TCC groups in the resting state. ^#^(*p* < 0.05) and ^##^(*p* < 0.001) indicate significant difference between the resting and movement states in the TCC group. ^&^(*p* < 0.05) and ^&&^(*p* < 0.001) indicate significant difference between the resting and movement states in the control group. ^$^(*p* < 0.05) and ^$$^(*p* < 0.001) indicate significant difference between the control and TCC groups in the movement state.

Figure 1 shows an example of the intensity or activation of the cerebral cortex in the resting and movement state of the both groups. Figure 2 shows the comparison results of wavelet amplitude (WA) between the TCC and control groups in different states. In the resting state, the TCC group showed significantly higher WA values in PFC in the five frequency intervals, MC and OC in intervals III to V, and LOC in interval II than those in the control group. Within the control group, the movement state showed significantly higher WA values in PFC in intervals II to V, and MC in intervals II to V than those in the resting state. Moreover, significantly higher values in LOC were found in intervals II to V in addition to ROC in intervals III, IV and V. Within the TCC group, the movement states showed significantly higher WA values in PFC in intervals II and III, LPFC in interval IV, MC in intervals II to IV, LMC in interval V, and OC in intervals II to IV compared with those in the resting state. In the movement state, the TCC group showed significantly higher WA values in PFC in intervals II and III, LPFC in intervals IV, MC in intervals III, LMC in intervals II, and OC in interval II and III compared with those in the control group.

### Wavelet phase coherence

**Figure 3:**
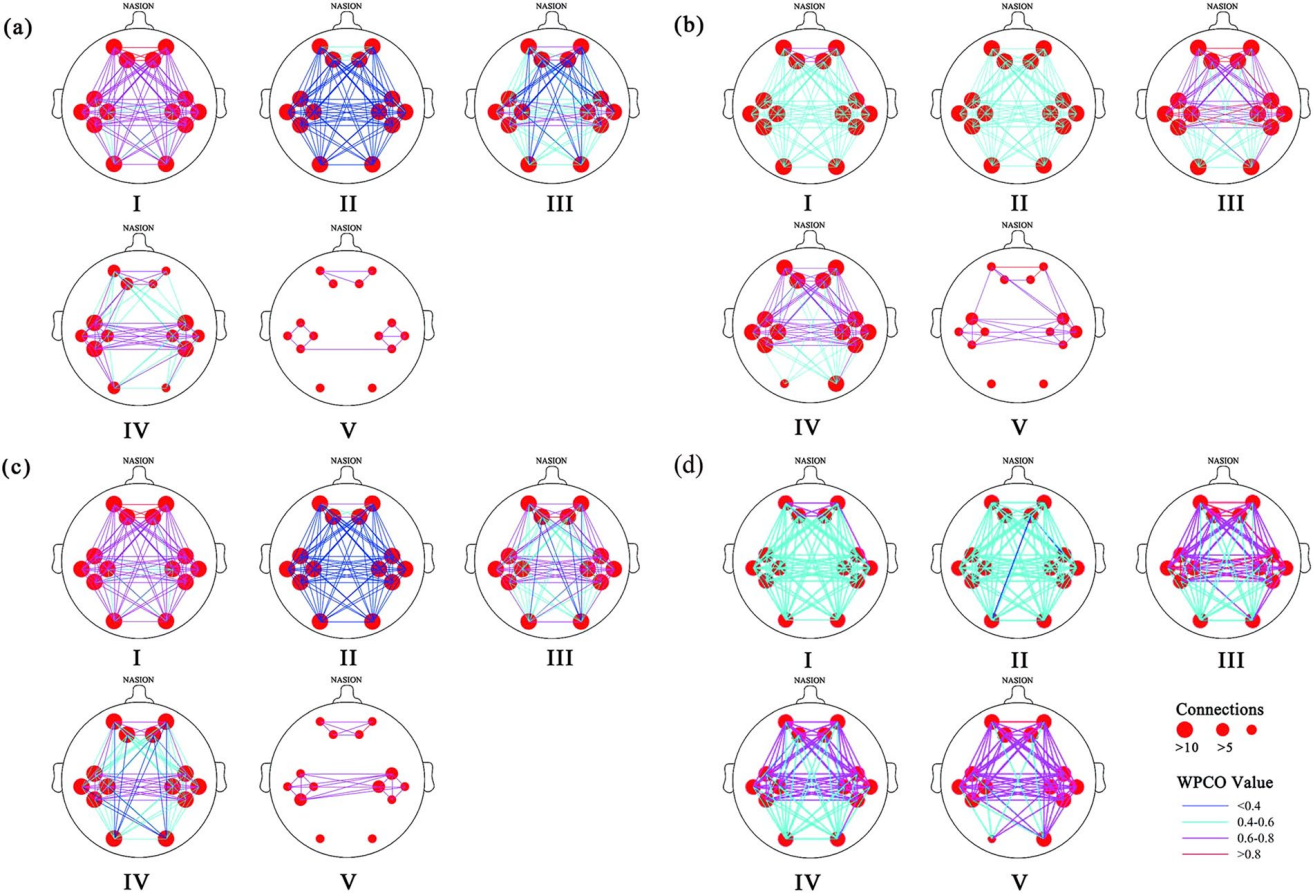
Frequency intervals I–V maps of the both group in different state. The control group at resting state (**a**), the control group at movement state (**b**), the TCC group at resting state (**c**), and the TCC group at movement state (**d)**. Connectivity line indicates a significant WPCO value between two channels. Line color indicates the connectivity intensity, and the sizes of the red dots indicate the numbers of connectivity among the channels.

Figure 3 shows the connectivity maps in the five frequency intervals under the different states in the TCC and control groups, and provideds a visual indication of the connectivity among the cerebral regions in each state. The line color indicates connectivity intensity, red dots indicate nodes, and the sizes indicate the numbers of connection among the channels. The connectivity was dense in intervals I to III and sparse in intervals IV and V; and increased connectivity was seen in intervals IV to V. Furthermore, connectivity was stronger in intervals II to V in the movement and resting states in the TCC group compared with the control group, however, connectivity was weak in interval I in the TCC and control groups during the movement state.

**Figure 4:**
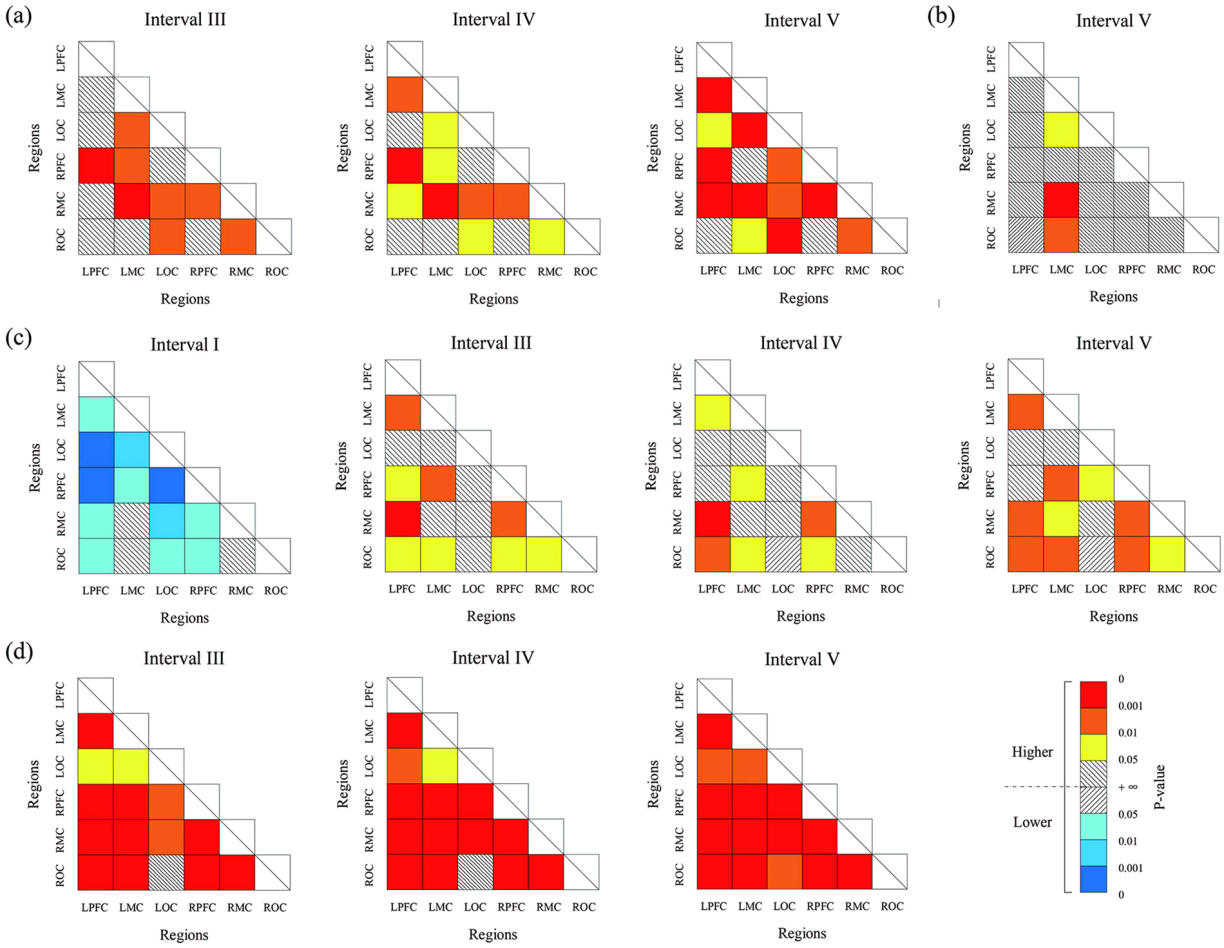
Comparison of WPCO values between the TCC and control groups in different state. TCC and control groups in the resting state (**a**), movement and resting states in control group (**b**), movement and resting states in the TCC group (**c**), and TCC and control groups in the movement state (**d**). Warm color represents the increased WPCO value; the brighter the color, the higher the significance. Cool color represents the decrease in WPCO value; and the deeper the color, the higher the significance.

Figure 4 shows the significant differences of WPCO values as revealed by the one–way ANOVA analysis. The WPCO value of LPFC–RPFC was significantly lower in interval I (F = 4.780, *p* = 0.034) in the resting state, but significantly higher in intervals III (F = 71.013, *p* < 0.001), IV (F = 42.263, *p* < 0.001), and V (F = 72.337, *p* < 0.001) in the TCC group compared with the control group as shown in Fig. 4(a). Moreover, the TCC group showed significantly higher values of LMC–LOC, LPFC–RPFC, LMC–RMC, LOC–RMC, LOC–ROC, RPFC–RMC, and RMC–ROC in interval III to V than the control group.

Figure 4(b) shows the significant differences of WPCO values between the movement and resting states in the control group and (c) shows the significant differences in the TCC group. The WPCO value of LMC–RMC was significantly higher in intervals III, IV and V. Moreover, in the control group, the movement state showed significantly higher values of LMC–LOC and LMC–ROC in interval V compared with the resting state. In the TCC group, the movement state showed significantly lower WPCO values in interval I in all cortical regions, except for LMC–RMC, LMC–ROC, and RMC–ROC, compared with the resting state. Furthermore, in the TCC group, the movement state showed significantly higher WPCO values in LPFC–LMC, LOC–RMC, and RMC–ROC in interval II, LPFC–RPFC and other eight channels in interval III, LPFC–RMC, and other channels in interval IV, except for LPFC–LOC, LPFC–RPFC, LMC–LOC, LOC–RMC, and LOC–ROC in interval V, compared with those in the resting state.

Figure 4(d) shows the significant differences of WPCO values between the TCC and control groups in the movement state. The TCC group showed significantly higher WPCO values in intervals III to V in all cortical regions, except for LOC–ROC in intervals III and IV than the control group.

### Coupling strength and direction

**Figure 5:**
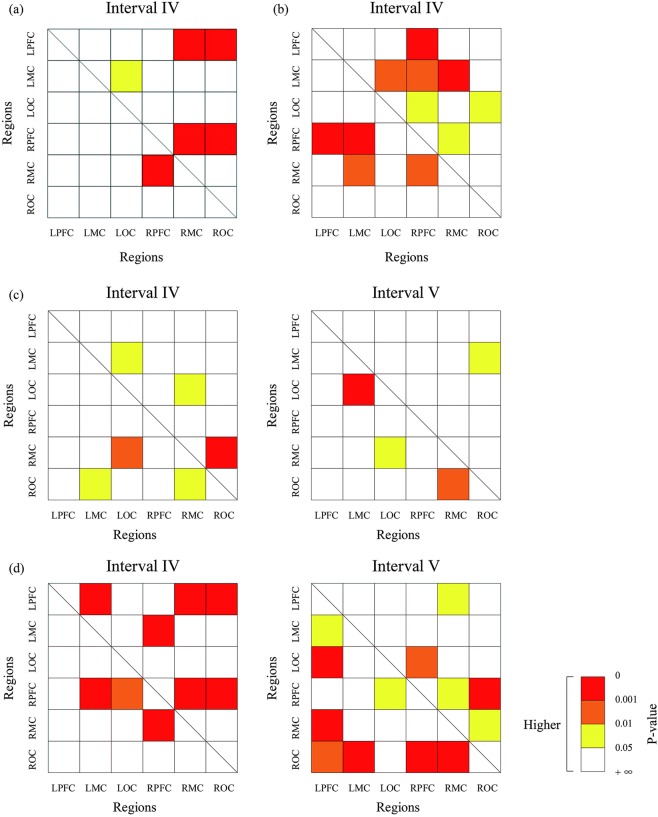
Comparison of the coupling strengths between the TCC and control groups in different state. TCC and control groups in the resting state (**a**), the TCC and control groups in the movement state (**b**), movement and resting states in control group (**c**), and movement and resting states in the TCC group (**d**). Matrix represents the significance of the coupling strength from the horizontal axis to the vertical axis. Red represents *p* value of 0–0.001, orange represents 0.001–0.01, yellow represents 0.01–0.05, and white represents no significance.

In the resting state, coupling strength was significantly higher in interval IV between RPFC and RMC in the TCC group compared with control group (*p* < 0.05). Moreover, significant coupling strength values were observed in ROC to LPFC, ROC to RPFC, RMC to LPFC, and LOC to LMC, as shown in Fig. 5(a). Additionally, in the movement state, significant enhanced coupling strength values were observed between LPFC and RPFC, RPFC and LMC, RPFC and RMC, and LMC and RMC in the TCC group compared with control group, as shown in (b). In the control group, comparison between the movement and resting states showed significantly enhanced coupling strength in LMC to ROC, LOC to RMC, RMC to LOC, and LOC to LMC in interval IV; and LMC to LOC, LOC to RMC, RMC to ROC, and ROC to LMC in interval V, as shown in (c). Similarly, in the TCC group, the comparison between the movement and resting states showed significantly enhanced coupling strength in LOC to RPFC, ROC to RPFC, and between RPFC and LMC, RPFC and RMC in interval IV. The same phenomenon occurred in interval V in LPFC to LMC, LOC and ROC; RMC to RPFC and between RPFC and LOC, RPFC and ROC, LPFC and RMC, as shown in (d).

**Figure 6:**
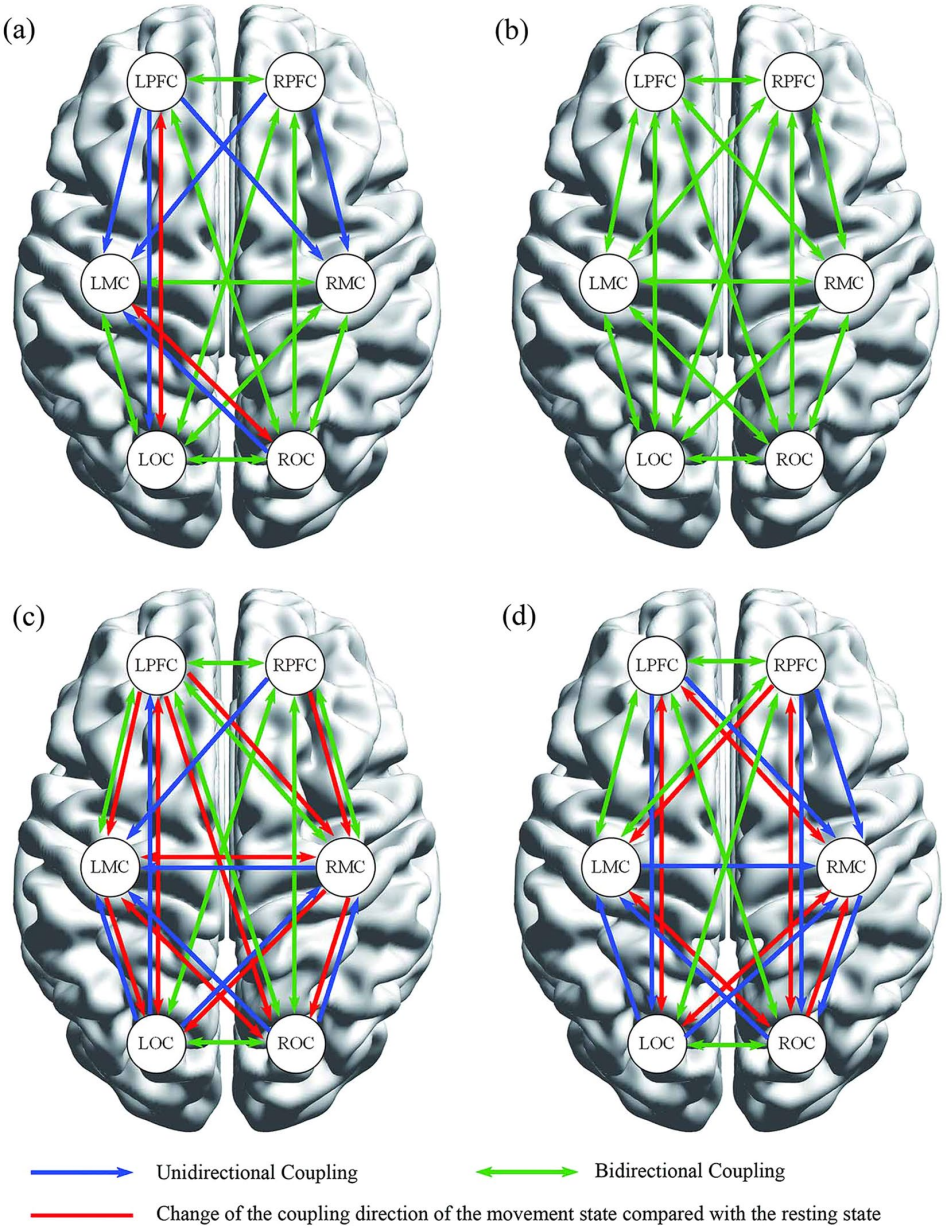
Coupling direction maps of the both group in interval IV and V. The control group at resting state and movement state (**a**), the TCC group at resting state and movement state (**b**) in interval IV, The control group at resting state and movement state (**c**), the TCC group at resting state and movement state (**d**) in interval V. Arrow direction indicates the effect direction, one–way arrow indicates unidirectional coupling, and double–headed arrow indicates bidirectional coupling.

In EC, the coupling relationships between cortical regions were divided into unidirectional and bi–directional couplings. Figure 6 shows the coupling relationship and directions of the resting and movement states in the TCC and control groups in interval IV. The PFC had a significant unidirectional coupling with the MC, LPFC with LOC, and ROC with LMC in the control group, whereas a synergistic effect was observed in other cortical regions in the resting state, as shown in Fig. 6(a). The red line shows the change of the coupling direction of the movement state compared with the resting state. The PFC had a significant unidirectional coupling with the MC and a synergistic effect was observed in the other cortical regions. The blue and green lines in Fig. 6(b) represent the coupling relationship and directions of TCC group in the resting state and the red line show the change of movement state compared with resting state. The synergistic effect with information transfer between brain regions was found in both states under the specific frequency interval IV. Figure 6(c) shows the coupling relationship and direction in interval V in the resting and movement states in both groups. The RPFC, RMC, ROC, and LOC had significant unidirectional coupling with the LMC in the resting state in the control group, which was similar to that in LOC and ROC with RMC and LOC with LPFC. The MC had unidirectional coupling with OC in the movement state of the control group. The significant unidirectional couplings of PFC, LMC with RMC, and OC with MC were found in the resting state of the TCC group. Moreover, LMC and RMC had unidirectional coupling with RPFC, LOC, and ROC in movement state of the TCC group as shown the red line in Fig. 6(d).

## Discussion

Cerebral fNIRS signals are mainly composed of evoked and non–evoked neurovascular couplings and systemic activity components^40^. Moreover, previous studies have demonstrated that the fluctuations of fNIRS signals in the five characteristic frequency intervals possibly reflect neurovascular couplings and systemic regulation activities. The sympathetic nervous system, endothelium–derived nitric oxide, and vascular myogenic responses are important in neurovascular coupling^41^. Beyond the intensity of activity in a particular cortical region induced by TCC, significant differences in FC and EC were observed between the TCC and control groups during resting and movement states. To our knowledge, this is the first study of the intrinsic functions of the human brain involving TCC practitioners in movement states, and reveals the effects of TCC practice on human brain function.

TCC is a mild to moderate aerobic exercise consisting of physical movement, meditation, and breathing as an interaction that aims to enhance the mind–body connection^42^. The WPCO in frequency interval I reflected the phase synchronization of cardiac activity in the brain. The brain redirects blood flow from other circulation districts to the cerebral circulation through the humoral and neural influence over the cardiovascular system. Cerebral blood flow (CBF) mechanism is the process of cerebral blood circulation re–integration. The corresponding cortical region activations accompanied by increased CBF is called functional hyperemia^43^. In our study, TCC exercise confers practitioners enhanced brain activation in the movement state as indicated by WA values. Enhanced activation indicates an increase in CBF, thereby significantly improving blood supply to the brain. Moreover, the decreased WPCO in interval I indicated a reduced coordinated regulation of cardiac activity to cerebral circulation in TCC practitioners at the movement state and this might affect substrate delivery and the removal of by–products of metabolism.

TCC moving meditation predominantly modulates the sympathetic nerves in PFC^44^. Recent neuroimaging studies have shown that long–term TCC practice could induce regional structural changes and influence the intrinsic functional architecture in the brain^8^, and meditation increases regional blood flow in the PFC. The total blood volume of the PFC is higher than the normal state due to the changes in oxyhemoglobin level^45^. This result is consistent with our study, wherein highly experienced TCC practitioners were significantly different from non–TCC practitioners in term of FC between PFC, MC and OC. TCC involves learning and memorization of new skills and movement patterns, and the complexity of the arrangement will affect the structure and function of the brain. For example, TCC increased brain derived neurotrophic factor and plasticity in brain morphology and function, including processes central to executive function^46,47^. In addition, meditation practice is a long–time process^48^. A study found that relatively three months of TCC practice could begin to improve memory performance in TCC naive elderly adults; thus, subjects without TCC practice do not immediately achieve benefits from meditation while practicing TCC. This might explains why the WPCO value of PFC was not significant in the comparison between the movement and resting states of the control group. However, due to the characteristics of TCC body movement, significance of LMC and RMC were found in myogenic activity, sympathetic nervous system of OC, and metabolic activities.

Frequency interval II is associated with respiration activity. The WPCO in frequency interval II did not show a significant difference in the TCC group movement state. This finding may be attributed to the stabilized breathing required by TCC. TCC is an exercise that combines slow motions with deep breathing, and practitioners are required to maintain a consistent breathing rate as much as possible, and meditation also has stabilizing breathing and heart rate effects. This suggested that during TCC training, the brain acquired continuous, deep and stable oxygen supply through the respiratory activity.

Frequency interval III is associated with myogenic activity and reflects the mechanical and electrical stimuli responding to smooth muscle regulation, which might originate locally from the myogenic activity of the smooth muscle cells of the vessels^49,50^ that are partly under autonomic control^22^. The enhanced connection among RMC with RPFC, LMC, and OC, and LMC with RPFC and OC in the TCC group proved that TCC practice increases the synchronization of myogenic activity.

The fluctuations in frequency interval IV, associated with neurogenic activities, are closely regulated through tight neurovascular coupling and partial autonomic control^51^. The dense synapses in cerebral vessels can release various vasodilatatory and vasoconstrictive factors, and cerebral vessels are regulated by the sympathetic nervous system, which can cause periodic contraction movement of these vessels^51^. The improved connection among LPFC–RPFC, LMC–RMC, LOC–ROC, PFC–RMC, and RPFC–LMC in the subjects with TCC practice suggested that TCC contributes to the enhanced synergy of nervous activities in bilateral brain regions.

Frequency interval V is associated with endothelial cell metabolic activities^25^. Endothelial cells can produce and release potent vasodilatatory factors such as nitric oxide and vasoconstrictive factors such as endothelin. Neuronal cells, glial cells, vascular endothelial cells, and other cells jointly compose the basic unit of neurovascular coupling effect, that is., the neurovascular unit^43^. Therefore, endothelial cell activities are closely connected with nervous activities. In interval V, significant connectivity was observed in bilateral brain regions. The elevated WPCO level between LPFC–RPFC, LMC–RMC, and LOC–ROC implied enhanced synchronation of metabolic activities in the left and right hemispheres, whereas the elevated WPCO values in LPFC–LMC, LPFC–LOC, and LMC–LOC pairs indicated enhanced anterior–posterior synergy in the brain.

Lateralization of brain connections has been shown to be a local property of brain networks in a previous study^52^. On the one hand, paying attention to stimuli involving language and encoding elicits brain activity that lateralizes to the left hemisphere in young individuals^53–55^. On the other hand, the stimulus associated with recall is right lateralized during, especially in the PFC^56^. Older adults experience reduced hemispheric asymmetry due to age-related deficits in neural connectivity^57^. For example, older adults had been found to recruit additional PFC regions during verbal recall, this change is reflective of a general aging phenomenon^58,59^. In this study, combining the results of frequency intervals IV and V, the TCC practitioners showed improved integration of nervous and endothelial metabolic activities in both hemispheres, which suggested that TCC could strengthen the network connection in the brain region of PFC and MC, and enhance the brain function. This result confirmed that TCC may help delay the brain neurological deterioration process.

Moreover, the WPCO of the TCC group movement state in interval III exhibited significant increase in LPFC–RMC, LPFC–ROC, LMC–RPFC, LMC–ROC, RPFC–RMC, and RPFC–ROC compared with the resting state. A similar case was observed in interval IV and V. The results indicated that myogenic, nerves and endothelial metabolic activities in the left and right hemispheres are characterized by increased integration during TCC exercise.

The WPCO values in intervals IV and V were significantly elevated in PFC–LMC and PFC–RMC, suggesting enhanced synchronization between PFC and MC during TCC exercise. Earlier studies have suggested that apart from being important in cognitive function, PFC accomplishes motion planning, organization, regulation, speed, and direction of motion^60,61^. PFC is a major hub of the default mode network^62^ and can combine information from the external environment with internal storage information^63^. Furthermore, the PFC controls top–down attention during conflict processing of alternative responses^64^ and is implicated in different aspects of social cognitive processing^65^ and undergoes changes with aging^66^. TCC includes training in sustained attentional focus, shifting, and multi–tasking which could help train working memory, divided attention, cognitive flexibility, and overall executive function^67^. In addition, evidence supports the communication between PFC and other brain regions, and this increased connectivity is associated with memory improvement^9,68,69^. Therefore, the result of this study further endorses the cognitive ability improvement and the anti–memory decline potential of TCC training^70–73^.

The changes in coupling strength represent the effect of one brain region to another. Neurogenic control of cerebral blood vessels is innervated by the sympathetic, parasympathetic and somatic sensory nerves on the brain surface, and is sufficient for regulating overall blood flow to the brain^74^. Hemodynamic parameters are closely regulated through tight neurogenic innervations and are under partial autonomic control in interval IV. Moreover, different postures lead to differences in hemodynamic responses in the cerebral and systemic vasculature, where autonomic reflexes are mediated by sympathetic, parasympathetic, and somatic sensory nerves^75^. In the present study, the MC network was dynamically reorganized, and the causal influence between PFC and MC in the TCC group was significantly greater than that in the control group during resting and movement states. Especially to deserve to be mentioned, the dynamic reorganization of the cortical network can contribute to balance control optimization^76^. This indicates that long–term practice of TCC enhances the control ability of the sympathetic and parasympathetic innervations in the PFC and MC; therefore, it may be one of the reasons for improving the balance of TCC practitioners.

Coupling direction reveals the information flow between brain regions^77^. An early study showed that frontal functions are susceptible to degenerative changes in normal aging^78^. Age–related shrinkage in gray matter volume and deterioration in cerebral white matter integrity are mainly distributed in PFC, this will gradually reduce the connectivity between the frontal–parietal areas, which are impaired in aging as shown from structural imaging^79^. In our study, the control group showed a large number of unidirectional coupling in interval IV and V, suggesting that PFC lacks drive–feedback mechanism in OC and MC in sympathetic, parasympathetic and endothelial metabolic activities, whereas, the TCC group exhibited increased bidirectional coupling during the movement state. These results indicated that the long–term TCC activity was beneficial to compensate for the neurogenic and endothelial metabolic activities of the PFC.

In our study, the main coupling direction in interval IV was on the left brain in the control group, especially regarding the effect on LMC. Simultaneously, this phenomenon occurred in the frequency interval V, and the coupling direction was from RPFC, RMC, ROC, and LOC to LMC. This phenomenon might be correlated with the habit of the right–handedness of the subjects, as a mechanism derived from the contralateral control of the brain^80^. However, the coupling direction in the TCC group during the resting state was from PFC, LMC, and OC to RMC, which was different from that of the left brain drive pattern in the control group. This suggested that the interactions among brain regions change after long–time TCC training.

Studies have proven that heart rate and respiration oscillations could be filtered out during analysis of fNIRS signals when estimating the evoked stimuli^81^; however, arterial pressure oscillations occurring spontaneously as Mayer wave existed in conscious subjects in the vicinity of 0.1 Hz frequency^82,83^. Mayer waves may affect the FC in interval III (0.052–0.145 Hz). Therefore, the interference of Mayer waves should be considered in future studies. Moreover, we have revealed Chen style TCC exercise related change in brain function. However, there are other forms of exercise similar to Chen TCC, such as Yang style TCC and Baduanjin, which have been found to be beneficial to human brain function^71^. Thus, it is valuable to compare the change in brain function of different style TCC by applying fNIRS during movement execution in the future.

## Conclusion

TCC is a form of exercise involving breathing, body coordination, visual perception and meditation that could induce activations of PFC, MC and OC, and improved blood supply to the brain. Significantly, there was less dynamic configuration of connection between brain regions in the short–term TCC training when the activation occurs; whereas, the long–term TCC training improved the connections of PFC, MC and OC in myogenic activity, sympathetic nervous system, and endothelial cell metabolic activities.

In the TCC movement state, the decreased synchronous contribution of cardiac activity to ΔHbO_2_ oscillations in the cerebral regions might affect substrate delivery and the removal of by–products of metabolism. After long–term tai chi training, the increased phase synchrony in the left and right hemisphere improves integration of nervous and endothelial metabolic activities in both hemispheres, this result confirmed that TCC may help delay the brain neurological deterioration process. Moreover, the increased connectivity between the PFC and other brain regions relay the ability of TCC training to improve cognition and the anti–memory decline potential.

The results of EC showed that long–term TCC training has a positive effect on the control ability of the sympathetic and parasympathetic innervations in PFC and MC. TCC training strengthened the bidirectional coupling between PFC and other regions in neurogenic and endothelial metabolic activities, which compensated for the driver–feedback mechanism. Intriguingly, the coupling direction of the brain was mostly on the left brain in the control group. By contrast, the unidirectional coupling on the right brain regions was more obvious in the TCC group compared with the control group. This suggests that the interactions among brain regions change after long–time TCC training.

## Materials and Methods

### Subjects

A total of 57 healthy right–handed subjects (28 females and 29 males, 60–70 years old) were recruited to participate in this fNIRS study; among which, 25 Chen style TCC practitioners (12 females and 13 males) and 32 controls (17 females and 15 males) were matched for sex, age, and education. According to the CONSORT guideline^84^, we calculated the minimum sample size was 20 in each group by analyzing the pre-experiment. All TCC practitioners have been practicing and teaching TCC for more than 5 years. The control participants with no TCC practice were recruited from local community as the non–sports population. Among the selected subjects, 2 were excluded because of loose detectors; thus, the valid number of sample used was 55.

Subjects were excluded based on the following criteria: (1) not right–handed; (2) with hypertension, systolic blood pressure ≥140 mm Hg or diastolic blood pressure ≤ 90 mm Hg; (3) with history of neurological or psychiatric diseases; (4) with drinking and smoking habits; (5) with brain trauma or had undergone brain surgery; (6) taking neurological or sleeping drugs; (7) sleep disorders; and (8) cognitive impairment.

The researchers introduced the basic information (including experimental purposes, procedures, schedules, announcements, and contributions) of the experiment to the study participants and obtained their consent. All participants were required to have adequate sleep and were not allowed to be involved in any strenuous physical activity (Moderate–intensity activity), such as running, mountain climbing, within 24 h before the experiment. We analyzed the basic information of the subjects by one-way ANOVA, as shown in Table 1. The experimental procedures were approved by the Human Ethics Committee of National Research Center for Rehabilitation Technical Aids and were in accordance with the ethical standards specified by the Helsinki Declaration of 1975 (revised in 2008). All participants provided written informed consent prior to participation.

**Table 1:** Participants basic information.

Parameters	TCC practitioners (N = 23)	Healthy controls (N = 32)	*P*
Age (years)	65.01 ± 2.61	65.34 ± 2.97	0.658
Gender (males)	12	17	NA
Body mass index (BMI)	22.66 ± 0.99	22.97 ± 0.89	0.548
Education (years)	12.29 ± 2.49	12.22 ± 2.43	0.899
TCC duration (years)	7.17 ± 1.59	NA	NA
TCC intensity (hour/week)	18.49 ± 2.45	NA	NA
Handedness (right)	23	32	NA
Systolic blood pressure (mmHg)	115.74 ± 12.73	122.47 ± 16.37	0.106
Diastolic blood pressure (mmHg)	71.57 ± 5.67	72.78 ± 6.59	0.478

### Procedures

Before the test, the subjects were required to sit for 10 min in a noiseless environment. The staff recorded the basic information, including sex, age, height and weight, blood pressure, disease history, and exercise duration, of the two groups of subjects on the day before the test. All subjects had a Montreal Cognitive Assessment Scale score of 26 or above, and the Pittsburgh Sleep Quality Index ranged from 1 to 6.

The both TCC group and control group experiments were divided into two states, namely, resting state and movement state. Each state lasted for 15 min, and another 10 min was allowed after the movement state to avoid the effect of fatigue. Before the experiment, the control group received a week–long TCC training to ensure their ability to complete the experimental process. During the resting state, subjects were asked to sit comfortably with eyes closed and remain quiet and in a sober state. The 56–form Chen–style TCC routine was used as the standard action in the movement state. It is a comprehensive competition routine created by the National Martial Arts Movement Management Center of China, and it is one of the most classic boxing routines in TCC exercise. The fNIRS was implemented continuously throughout the experiment.

### Functional near–infrared spectroscopy

**Figure 7:**
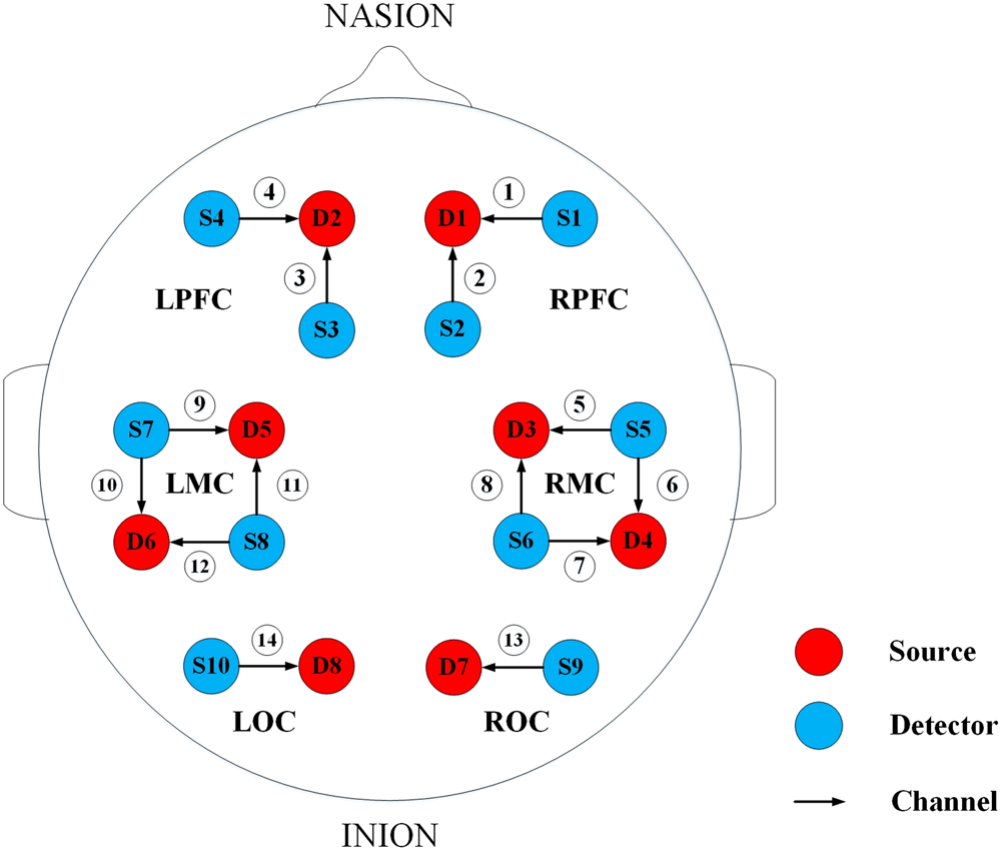
Position of the detector and light source in the six cerebral cortices. Red circle represents the detector, blue represents the light source, and the arrow is the channel of the light source pointing to the detector.

A technology multi–channel tissue oxygenation monitor with continuous-wave (NirSmart, Danyang Huichuang Medical Equipment Co., Ltd) was used in fNIRS measurements. Each sensor of the instrument consisted of a three –wavelength light emitting diode, which served as the source optode and emitted light at wavelengths of 740, 808 and 850 nm, and a detector optode. The inter–optode distance was 30 mm. We initially set all differential path-length factors to 7.0. The instrument measured the raw light intensity signals. Based on different absorption spectra, concentration changes of HbO_2_ were calculated from the changes in detected light intensity using the modified Lambert Beer law, assuming constant scattering. 14-channels of the fNIRS were symmetrically positioned over the regions of the left and right PFCs (LPFC/RPFC), MCs (LMC/RMC) and OCs (LOC/ROC). The distribution of channels in the bilateral motor area evenly covered the electrode sites Fp1/2, FC1/2, FC3/4, CP1/2, CP3/4, O1/2. The calibration function of the instrument and the corresponding template were used to ascertain the channels to fill exactly in correspondence of the 10/10 electrode positions according to different head sizes. Figure 7 shows the distribution of 14 measurement channels, consisting of 10 light source probes and 8 detector probes, in the brain. Signals with a low signal–to–noise ratio were removed, and the sampling rate was 10 Hz.

### Data pre–processing

The pre–processing method of fNIRS data had been elaborated in our previous studies^85–87^. All data processing was performed by using MATLAB (version 2017b). First, the moving average method was used in order to eliminate the obvious abnormal points in the signal. The time window used for the moving average filter was 3 s. The processing method based on moving standard deviation and cubic spline interpolation was applied to remove motion artifacts. The artifact portion was determined by identifying the sliding window standard deviation above a certain threshold and was removed by cubic spline interpolation. Then independent component analysis (ICA) analysis was then performed on Delta [HbO_2_] signals of each channel to reduce physiological interference in fNIRS measurements, including cardiac pulsations, respiratory signals, and blood pressure changes. All the ICA-derived components were visually inspected to determine the components that might be related to noise and artifacts. Finally, we used a six-order Butterworth band-pass filter to obtain the filtered signals of 0.005–2 Hz with an improved signal-to-noise ratio and to remove super–low–frequency elements (below 0.005 Hz) of the raw signal. The purpose of signal pre–processing is to remove the outliers and trend terms of the overall signal and thus facilitate the subsequent analysis.

### Functional connection analysis

The FC was calculated using wavelet–based coherence method, which had been described in our previous studies^88–91^. Wavelet transform (WT) is a transformation method of time series from the time domain to the frequency domain to obtain the main component of the time series in the frequency domain. Tunable filter band lengths were used to provide the appropriate time and frequency resolution^92^, which projected the time series onto the time–frequency plane, thereby obtaining the time–frequency–amplitude 3D map. Morlet wavelet has its best time–frequency compactness^93^ and thus was used for continuous WT in this study. The results of WT were averaged over the time domain to obtain the WA of each Delta [HbO_2_] signal at each time and frequency. WA is characterized by the intensity or activation of the cerebral cortex.

The WPCO is a method of using the phase information of the signal to evaluate the correlation of two signals, which quantitatively represents the instantaneous phase of the two signals at a consistent degree throughout the continuous process of the time series to identify possible connectivity^94^. The WPCO value was between 0 and 1, which characterizes the instantaneous phase difference between the two Delta [HbO_2_] signals as a constant trend throughout the study. The high WPCO value indicates that an agreement between the two cortical regions exists, otherwise it indicates that less relationships between the two Delta [HbO_2_] signals exist^95^.

In order to identify significant coherence, the amplitude–adaptive Fourier transform (AAFT) method was applied to perform WPCO test^94^. A total of 50 surrogate signals were produced, which possess the same mean, variance, and autocorrelation functions as the original signal but without any phase correlation. The phase coherence level of the original signal was verified by calculating the phase coherence between the substitute signals. This study has calculated the WPCO values between the two original Delta [HbO_2_] signals, the WPCO mean, and the two standard deviations above the mean of the surrogate signal. The connectivity in the frequency interval was considered significant when the WPCO value of the original signal was higher than two standard deviations above the mean of the surrogate signal^96^.

### Effective connection analysis

EC characterizes the interdependence of brain regions. The different degrees of master–slave coupling existed in various brain regions in the cerebral oxygenation signals. EC was characterized by the coupling strength and coupling direction of the brain region from the phase coupling. The coupling strength indicates the degree of influence of a brain region on another brain region, and the coupling direction reveals the source of the brain region^97^.

Through the dynamic Bayesian inference, which is a method of the priori event model that is used to estimate the posterior event model distribution, the coupling coefficient matrix *c* between two time series can be obtained according to the measured time series^98^. The coupling strength can be calculated by the coupling function *q*. The coupling strength between the oscillators Φ_*i*_ and Φ_*k*_ is defined as the Euclidean norm of the coupling coefficient matrix^97^:

1 $$\Vert {q}_{i\to k}\Vert =\sqrt{\sum {({c}^{(i:k)})}^{2}}$$

In order to distinguish the driving direction between brain regions, the coupling direction is defined as:

2 $${D}_{i,k}=\frac{{q}_{i\to k}-{q}_{k\to i}}{{q}_{i\to k}+{q}_{i\to k}}$$

In this case, *D*_〈*i,k*〉_ ∈ [−1, 1], and if *D*_〈*i,k*〉_ is the regular expression Φ_*i*_ drives Φ_*k*_, whereas the other direction is Φ_*k*_ points to Φ_*i*_. This study mainly focused on the study of EC in IV and V frequency bands to analyze the brain interval drive and feedback relationship between neural activity and endothelial cell metabolism. Similarly, the study used AAFT to generate 50 substitute signals to test the EC significance.

### Statistical analysis

The normal test (Kolmogorov–Smirnov test) and variance uniformity test (Levene test) of each subject’s data were performed at the group level to ensure that the assumptions required for parameter analysis were satisfied. In this study, one-way ANOVA was performed on the channel-wise WA, WPCO and EC in the resting and movement states between the Chen style TCC and control groups at the specific frequency interval. Bonferroni correction was used for the multiple comparisons. The corrected p-value threshold was set at *p* < 0.05.

## Data Availability

The datasets generated during and/or analysed during the current study are available from the corresponding author on reasonable request.
